# Eutectic mixture of local anaesthetics (EMLA®) as a primary dressing on painful chronic leg ulcers: a pilot randomised controlled trial

**DOI:** 10.1186/s40814-018-0312-6

**Published:** 2018-07-07

**Authors:** Anne Purcell, Thomas Buckley, Jennie King, Wendy Moyle, Andrea P. Marshall

**Affiliations:** 1 Menzies Health Institute, Griffith University, Nathan Campus, Building N48, Room 2.10, 170 Kessels Road, Nathan, Queensland 4111 Australia; 2 School of Nursing and Midwifery, Griffith University, Nathan Campus. Building N48, Room 2.06, 170 Kessels Road, Nathan, Queensland 4111 Australia; 3 Central Coast Local Health District, Gosford Hospital, Holden St, Gosford, New South Wales 2250 Australia; 4 Sydney Nursing School, University of Sydney, 88 Mallett St, Camperdown, New South Wales 2050 Australia; 5 Gold Coast University Hospital, Gold Coast Hospital and Health Services, E. 2 015, 1 Hospital Blvd, Southport, Queensland 4215 Australia; 6 National Centre of Research Excellence in Nursing, Room 2.08, G01, Griffith University, Southport, Queensland 4222 Australia

**Keywords:** Chronic leg ulcers, Wound-related pain, EMLA®

## Abstract

**Background:**

The physical, occupational, social and psychological impact of chronic leg ulcers (CLUs) on an individual is considerable. Wound-related pain (WRP), the most common symptom, is frequently reported as moderate to severe and mostly occurs at dressing change. WRP pain may not be alleviated by oral analgesics alone. Persistent poorly controlled leg ulcer pain can negatively impact wound healing and health-related quality of life (HRQoL).

**Methods:**

A pilot, parallel group, non-blinded, randomised controlled trial was conducted in six procedure clinics located in a public community nursing service in New South Wales, Australia to evaluate eutectic mixture of local anaesthetics (EMLA®) on painful CLUs when used as a primary dressing. The primary objective was to assess feasibility by using pre-determined criteria: at least 80% recruitment rate, 80% retention rate and 80% adherence to the study protocol. Key eligibility criteria were that participants had a painful CLU no larger than 100 cm^2^, a numerical rating scale (NRS) wound-related pain intensity score equal to or greater than 4, low to moderate exudate, no contraindications to EMLA® and capacity to consent. One hundred and seven patients with painful CLUs were screened for eligibility; 56% (*n* = 60) were eligible and consented to participate in the study. Participants were randomly assigned to the intervention (*n* = 30) or control (*n* = 30) groups. The intervention group received a measured dose of the topical anaesthetic EMLA® 5% cream daily as a primary dressing for 4 weeks followed by usual wound management for a further 8 weeks. The control group received usual wound management. Participants and investigators were not blinded to the treatment. WRP was measured at every dressing change. Wound healing and HRQoL were measured at baseline, 4 and 12 weeks.

**Results:**

Recruitment rate was lower than expected which likely meant patients were missed. Study retention rate was 90% (*n* = 54). Intervention fidelity was impacted by availability of resources and patient factors such as increased WRP.

**Conclusion:**

This study identified that a larger randomised controlled trial investigating EMLA® applied as a primary dressing on painful chronic leg ulcers is feasible with modifications to the study protocol.

**Trial registration:**

Australian New Zealand Clinical Trials Register: Registered 16 December, 2009

## Background

Wound-related pain (WRP) is the most common symptom of CLUs with reported prevalence as high as 85% [1, 2]. The most significant pain occurs at dressing change [3, 4]. For many individuals, WRP persists despite the use of conventional pharmacologic strategies such as oral analgesia [4]. Topical analgesia and anaesthetics applied directly to the wound bed is an option for relieving WRP [5]. The eutectic mixture of local anaesthetics cream (EMLA®) has been shown to be effective for relieving pain that occurs during debridement of CLUs [5]. Regulations for the use of EMLA® on open wounds such as CLUs and its drug schedule status may differ between countries. High quality evidence evaluating topical anaesthetics for managing WRP is still emerging [5–7].

In our published single-case report [8], we suggested that the topical application of EMLA® as a primary dressing may be a promising therapy for managing pain associated with CLUs but recognised that this treatment was yet to be empirically tested. Additionally, most individuals with painful CLUs are older with multiple comorbidities so higher rates of non-compliance are more likely. Therefore, in line with current recommendations, we conducted a pragmatic, external pilot, parallel group, randomised controlled trial (RCT) to evaluate the protocol implementation which would inform a larger trial [9]. A pragmatic approach was selected to assess the potential effectiveness of the intervention, the secondary objective, in a routine real-life practice setting. To assess feasibility as the primary outcome of this study, we were guided by the feasibility framework developed by Thabane et al. [10] including the following:

(1) Eligibility, recruitment and retention; (2) resource requirements; (3) human resources and data management; and (4) scientific assessment to identify potential effectiveness and any adverse events resulting from the intervention [10].

Study feasibility was assessed using the following pre-determined criteria for determining success:

- Recruitment of at least 80% of eligible patients within 12 months;
- Retaining 80% of participants in the study;
- Achieving 80% adherence to the intervention protocol

This paper reports on the feasibility findings of this pilot study. Patient-related outcomes were evaluated in this pilot study as secondary outcomes with the findings reported elsewhere [11, 12].

## Methods

### Study design

**Fig. 1:**
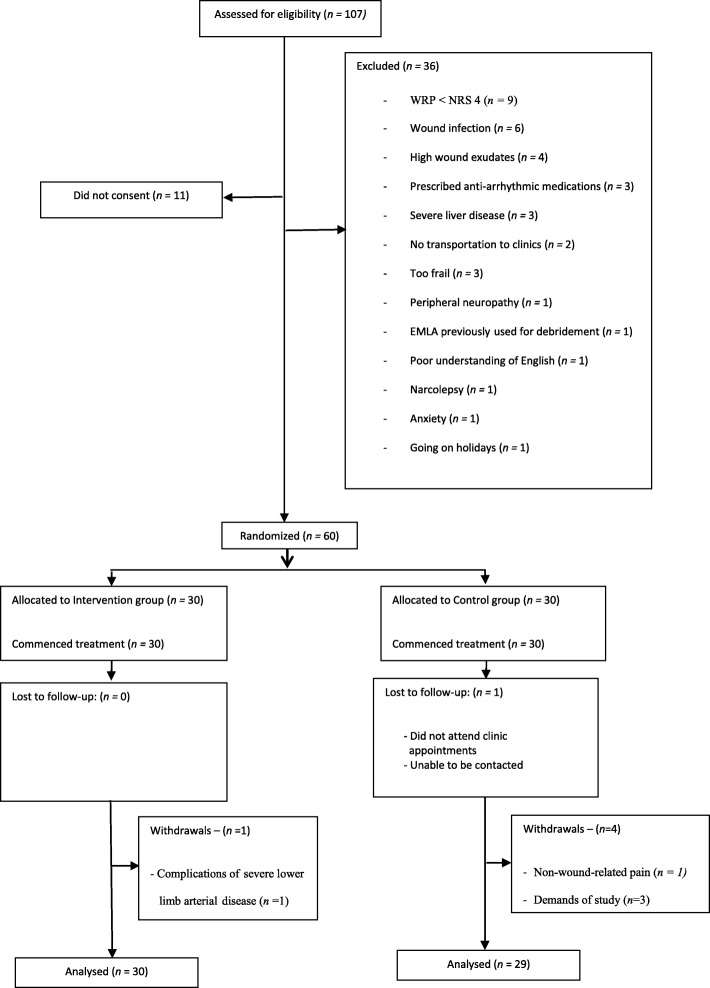
Flow of participants through study

This feasibility study was a pilot, parallel group, non-blinded, randomised, controlled trial (RCT). The study protocol was approved by the Northern Sydney Health (AU RED Ref. HREC/09/HARBR/162) and the Griffith University Human Research (GU Ref No: NRS/16/12/HREC) Ethics Committees (HREC), registered with the Australian New Zealand Clinical Trials Register (ACTRN12609001080213) and conducted in accordance with the Declaration of Helsinki (revised 2013); written informed consent was obtained from all participants. The study is reported according to the CONSORT 2010 statement [13] (Fig. 1). In line with Good Clinical Practice (GCP) [14], complications such as adverse reactions to EMLA® or wound infection were reported to the Data Safety and Monitoring Board (DSMB) and HREC.

### Setting and sample

Participants considered for inclusion in this study were individuals already referred to a large health district community nursing service in New South Wales, Australia. This study was conducted across six procedure clinics within the service where approximately three-quarters of patients required wound management; 76% had one or more CLUs [15].

Community nurses assisted with preliminary screening of patients and notified the study investigators of any patient with a lower leg ulcer greater than 6 weeks’ duration, who required analgesia for WRP and had the capacity to attend the community nursing clinics for wound management. Potential patients were assessed against the inclusion and exclusion criteria (Table 1) by a member of the research team. Eligible patients were informed of the study and consent obtained.

**Table 1:** Inclusion and exclusion criteria and rational for inclusion in study

	Rationale
Inclusion criteria	
1. One or more chronic lower leg ulcer(s) of at least 6 weeks duration	A chronic wound is defined as one which has not followed the expected path of healing when related to time, appearance and responses to optimum wound management and is often demonstrated when a wound has not shown signs of healing within a 6-week period [54]
2. Wound size up to 100 cm^2^ in size in total	The maximum wound size of 100 cm^2^ was selected due to the maximum dose/surface area ratio of 1–2 g EMLA® to 10 cm^2^ of wound surface area, the recommended maximum dose being 10 g. This dose/surface area ratio is recommended for the application of EMLA® when used for the debridement of non-viable tissue from a leg ulcer [55]
3. Patients with low to moderate wound exudate	This will enable the EMLA® to remain on the wound bed over 24 h and not run off the wound due to excessive wound exudate
4. Numerical rating scale (NRS) pain score ≥ 4 at assessment and/or within the previous week	WRP ≥ NRS of 4 can indicate uncontrolled pain during or after dressing change which may require a change of management [56]
5. Patients currently requiring oral analgesics due to previously reported wound-related pain	Individuals with WRP are often prescribed oral analgesia indicating their level of pain is significant
6. Patients ≥ 18 years of age	The prevalence of CLUs increases with age thus the prevalence in those < 18 years of age is low [57]
7. Patients with the capacity (cognition and/or language)	Participants in this study should be capable of providing well-informed and considered consent
8. Patients have the capacity to attend the CCCNS Procedure Clinics. The exception is participants requiring visits on weekends and public holidays; health centres are closed at these times. This was in accordance with the NSCCH Home Safety Procedures for Community Nursing, CCH (PR2007_016).	Continuity of care
Exclusion criteria	
1. Patients scheduled for leg amputation	Amputation of the lower limb with a CLU would negate the need for wound management
2. History of peripheral sensory neuropathy (PSN) of the feet Modification: After 6 July, 2011, patients with painful peripheral neuropathy were no longer excluded	This study includes painful CLUs. Individuals with PSN often do not experience any peripheral sensation in their feet or lower legs [58] Patients with painful peripheral neuropathy can also experience painful WRP
3. Patients that have had or require the use of EMLA® for debridement of the wound bed within the previous 4 weeks before recruitment Modification: After 9 February 2011, patients were only excluded if they had EMLA® applied for debridement of the wound bed within the previous week	The introduction of EMLA® within 4 weeks may influence baseline data The half-life lignocaine and prilocaine is 65–150 and 10–150 min, respectively, a similar half-live of their intravenous counterparts [55, 59]
4. Patients with suspected wound malignancy or pyoderma gangrenosum confirmed by biopsy	The management of leg ulceration caused by malignancy or pyoderma gangrenosum requires different management than ulcerations due to venous, arterial or diabetic aetiology
5. Patients with diagnosed localised or spreading clinical wound infection	Management of wound infection requires the introduction of strategies that may influence the individual’s pain levels beyond the capabilities of the primary dressing
6. End-stage palliative care patients	End-stage palliative patients often require increased use of analgesics for palliation, thus the effectiveness of an intervention may be masked
7. Patients where EMLA® is contraindicated or cautioned	To reduce potential participant harm [55]

*WRP* wound-related pain, *HRQoL* health-related quality of life, *CLU* chronic leg ulcer, *EMLA®* eutectic mixture of local anaesthetics, *NRS* numerical rating scale, *CCCNS* Central Coast Community Nursing Service, *CCH* Central Coast Health, *PSN* peripheral sensory neuropathy

A sample size of 60 was selected for this study as it was thought to be a good representation of the target population, large enough to provide practical information about the feasibility aspects of the study [10]. Although reported sample sizes for pilot studies vary, the median sample size per arm is often 30 [16]. In an external pilot study such as this, there is a ‘trade-off’ between maximising the precision of estimates of important parameters and sample size which impacts resources, time and costs of a study [17]. To address feasibility, we enrolled 60 patients to accommodate possible attrition throughout the 12-week study period. Attrition rates are often high in wound studies due to comorbidities associated this patient group particularly when the study period is long, where wounds may deteriorate, and the innate difficulty to adhering to the protocol over many weeks [18]. Even so, based on the community service patient profile, we estimated it would take approximately 12 months to enrol 60 patients assuming a 10% drop out rate [19]; a 20% drop-out is considered acceptable [20].

### Randomisation, blinding and allocation concealment

Following eligibility assessment and consent, a simple randomisation method (1:1) was used to randomise participants to either the intervention (EMLA®) or control group. PASS 2008 Power Analysis and Sample Size software (NCSS, Kaysville, UT) was used to generate the allocation sequence by a researcher not involved with screening patients; the investigator was blinded to the allocations. Patients were allocated to study groups by retrieving the next in a series of sequentially numbered, opaque, pre-prepared sealed envelopes. This method for allocation concealment can achieve a low risk of bias [21].

Similar to other wound care studies [18], it was not possible to blind the participants, treating nurses or the investigators to the treatment allocation as the intervention was compared to usual care and not a placebo. However, the statistician was blinded. To minimise bias, the intervention and control groups were treated as equally as possible apart from the intervention itself. Treating clinicians were required to adhere to the Australian Standards for Wound Management [22] and health service policies and procedures.

### Interventions

An intervention period of 4 weeks was selected as healing rates over a 4-week period can determine intervention effectiveness [23–25]. Patients were followed-up over a 12-week period because wound margin advancement, initial healing rate and percentage of wound surface area reduction are strong indicators of complete healing at 12 weeks independent of topical dressing used [25].

#### Intervention group

Participants in the intervention group received a measured dose of EMLA® daily as a primary dressing to their CLUs for 4 weeks followed by 8 weeks of usual care. EMLA® (Aspen Pharmacare, St Leonards, New South Wales, Australia) is a non-sterile, preservative-free, eutectic mixture of two amide type local anaesthetics lignocaine 2.5% and prilocaine 2.5% and has a good safety profile when used for debridement [26–28]. The EMLA® dose of 1–2 g per 10 cm^2^ to a maximum dose of 10 g was based on manufacturer recommendations [28]. At each dressing change during the intervention period and following wound cleansing, EMLA® was drawn up into a syringe then spread evenly over the wound bed.

To ensure high quality and consistent application of the EMLA®, we developed an intervention protocol and provided education sessions and supervision of nurses to promote intervention fidelity. All treatments were performed by community nurses with previous experience in wound management. To assess the timing and dose of the intervention delivery and effect of the intervention on participants, random quality assurance visits to clinics or patient’s homes, review of the participant’s medical records and follow-up of the data collected were made by the chief investigator.

#### Control group

Initially, we considered using a placebo as the control group intervention however, further consideration identified that a placebo would require daily dressing changes to enable a placebo cream to be applied. The daily dressing change would confer no benefit to the participant and had potential to negatively impact wound healing; such an approach was difficult to ethically justify. Participants in the control group received usual care for 12 weeks where the primary dressing and dressing frequency (daily to weekly) were determined by clinical judgement over the treatment period. Usual care primary dressings could change throughout the study period in line with changes in exudate, non-viable tissue and microorganism levels over the course of treatment. Most common primary dressings used were hydrofibre, hydrogel, enzyme alginogel, povidine iodine mesh, cadexamer iodine and silver impregnated dressings.

Both groups received the same secondary dressing, a soft non-woven, hydrophobic polyamide fibre containing cellulose fluff core (Zetuvit®) [29]. Participants with low exudating CLUs in the intervention and control groups who experienced an increase in WRP during dressing change from secondary dressing adherence had a triglyceride mesh applied over the primary dressing and under the secondary dressing to prevent adherence. All wounds were cleansed with normal saline 0.9%. If clinically indicated, conservative sharp wound debridement and compression therapy were implemented. Regardless of treatment allocation, and in line with standard practice, all participants received EMLA® prior to conservative sharp debridement to eliminate operative pain. EMLA® was removed prior to debridement. Intervention group participants had EMLA® reapplied following debridement.

### Management of adverse events

Participants recorded any problems or adverse reactions to the intervention in their Pain Diary which was reviewed at each clinic visit. Treating clinicians observed for known reactions to EMLA® such as blanching, erythema, oedema, pruritus, burning, purpura and contact hypersensitivity [28] at each dressing change and documented them in the participant’s electronic medical record. Additionally, a Notification of Adverse Event form was completed followed by immediate notification and discussion with the chief investigator (CI). In the event of any adverse reaction in the intervention group, treatment was ceased immediately and the medical team notified. Notifications of adverse events were mandatory and reported promptly to the NSH HREC and the DSMB by the CI.

### Measurements used to address study aims

The feasibility of conducting an RCT in a public community health service to prevent unnecessary spending or wasting of resources in a larger study was measured by assessing quantitative and qualitative data to address key study processes, resources, management and scientific feasibility. Quantitative feasibility outcome measures included validation of the recruitment rate and randomisation processes, consent rate, retention rate, and suitability of the eligibility criteria and, data collection instruments. Additionally, to measure intervention adherence, the number of protocol deviations or violations during the study by treating nurses and participants were identified, and the reasons why these occurred were documented. Further quantitative and qualitative measures included the availability and commitment of human resources, the time to perform study processes, availability and quality of study equipment, data management outcomes and challenges and the cost estimates to conduct such a study. The feasibility of the intervention was measured by evaluating the patient’s physiological responses to the intervention and the monitoring of adverse events. Qualitative measures included participant and investigator burden. Scientific feasibility was assessed to address the clinical responses to intervention. Scientific feasibility is reported elsewhere [11, 12].

Additional, patient-specific data were collected. Baseline measurements are detailed in Table 2. Data collection instruments, their psychometric properties, assessment time-points and estimated time to complete are presented in Table 3. When data specific to a leg ulcer were required and if a participant had more than one CLU, the largest ulcer was the reference ulcer; all ulcers were treated as per group allocation.

**Table 2:** Baseline measurements

Participant history	Wound-related pain	HRQoL relating to CLU	CLU Characteristics
- Socio-demographic history - Medical and surgical history	- WRP at dressing change -Before -During -After - Pain type - Quality - Location - Triggers - Relievers - Pain medications - Effects on activities	- Social life - Wellbeing - Physical symptoms - Overall HRQoL [60]	- Leg ulcer surface area - Aetiology and duration of leg ulcer - Ankle Brachial Pressure Index - Leg ulcer measurement: -Exudate type and amount -Necrotic tissue type and amount -Granulation type and amount -Condition of wound edges -Peri ulcer viability - Oedema type & location - Assessment of bioburden - Wound-related pain intensity and frequency assessment over previous 24 h - How HRQoL relates to the leg ulcer [61]

*WRP* wound-related pain, *HRQoL* health-related quality of life, *CLU* chronic leg ulcer

**Table 3:** Data collection instruments

Data collection instrument	Outcome measure	Estimated time of completion	Psychometric properties	Outcome assessment time points
11-point numerical rating scale [62, 63]	Wound-related pain intensity	1 min	Discriminative power relating to chronic pain. Test, re-test reliability high (*r* = 0.96); Construct validity—highly correlated with the visual analogue scale for chronic pain conditions (reported range, 0.86 to 0.95) [64]	Baseline and every dressing
Cardiff Wound Impact Schedule [60]	Health-related quality of life	5 to 10 min	Established reliability with internal consistency subscale scores > 0.75, and good reproducibility [60]	Baseline, 2, 4, 8 and 12 weeks
Leg Ulcer Measurement Tool [61] -Clinical and patient domains	Chronic leg ulcer appearance	5 to 10 min	Concurrent construct validity high (*r* = 0.82) with excellent intra-rater/inter-rater reliability for the total LUMT scores (ICC > 0.75) and for many of the 14 domains; some domains were less reproducible; this tool was able to detect change in wound status over time [61]	Baseline, 2, 4, 8 and 12 weeks
Wound-related pain at dressing change assessment tool [56, 65]	Wound-related pain response to intervention over the previous 24 h	5 min	Data collection tool not validated	Baseline and every dressing
Wound photography and 2-dimensional photo-digital planimetry [66]	Chronic leg ulcer measurement over time (cm^2^)	15 min including download onto computer for measurement	Inter-rater reliability and intra-rater reliability is higher than traditional wound tracing methods (94 and 98.3%, respectively) [66]	Baseline, 2, 4, 8 and 12 weeks
American Geriatric Society Pain Diary [67]	WRP intensity and frequency of pain-relieving medications, mood and response to wound treatments [68, 69]		Data collection tool, not validated	Whenever pain perceived at home

*ICC* intraclass correlation coefficient, *r* correlation coefficient, *LUMT* Leg Ulcer Measurement Tool

### Data analysis

Data were entered and checked for missing and invalid values in Microsoft Excel® then imported into Statistical Analysis for Social Scientists (SPSS Version 22, Chicago, USA) for analysis. A random sample (10%) of the data was verified against the original case report form. Quantitative data were analysed using descriptive statistics. Qualitative data from field notes were analysed using descriptive content analysis [30].

## Results

Detailed results for key components of the feasibility assessment are provided below.

### Study process assessment

#### Recruitment

Participants considered for inclusion in this study were individuals already referred to a large health district community nursing service New South Wales, Australia. We screened 107 patients with painful CLUs of whom *n* = 70 (65%) were eligible. Sixty of the eligible patients (56%) consented to participate in the study. While this met the feasibility objective, the recruitment rate was slower than anticipated and took 30 months (September 2010 to March 2013). In total, 30 patients screened did not meet the eligibility criteria. The most common reasons patients were excluded were insufficient wound-related pain, the presence of wound infection and high wound exudate. Twelve of the screened participants were not eligible due to frailty and chronic disease; seven of these participants were excluded due to the exclusion criteria. In the first 5 months, only three patients were recruited. We recognised that the exclusion criteria were too restrictive and modification took place to improve recruitment rates (Table 1). In addition, the appointment of a research assistant (RA) to assist the chief investigator (CI) also increased the recruitment rate to 2–3 per month. Comparison of groups’ socio-demographic and clinical history at baseline is presented in Table 4. Completed follow-up of all participants took 33 months (September 2010 to June 2013). The trial ended once follow-up for all participants was complete.

**Table 4:** Comparison of Groups’ socio-demographic and clinical history at baseline

	Intervention group (*n* = 30)	Control group (*n* = 30)	
	Mean (SD)	Mean (SD)	*P*
Age (years)	73.4 (12.5)	73.8 (10.1)	0.89
CLU duration (weeks)	26.4 (26.0)	20.5 (13.4)	0.32
CLU surface area (cm^2^) at baseline	8.01 (10.4)	9.2 (8.9)	0.48
Sex	n (%)	n (%)	
Male	13 (43.3)	12 (40.0)	0.79
Female	17 (56.6)	18 (60.0)	0.79
Ulcer type			
Venous	18 (60.0)	22 (73.0)	0.27
Arterial	5 (20.0)	3 (10.0)	0.45
Mixed	5 (13.3)	3 (10.0)	0.45
Incompressible	1 (3.3)	2 (6.6)	0.55
Diabetic foot ulcer	1 (3.3)	0	0.31
Pain medications			
Opiates	17 (56.6)	13 (40.0)	0.30
NSAIDS	4 (13.3)	5 (16.6)	0.72
Other pain meds	21 (70.0)	23 (76.6)	0.56

*SD* standard deviation, *p p* value, *CLU* chronic leg ulcer, *NSAIDS* non-steroidal anti-inflammatory drugs

#### Retention

A retention rate of 90% of study particpants was achieved. One patient in the intervention group and five in the control group did not complete the study (Fig. 1). One participant required a below knee amputation, another withdrew due to severe back pain and three withdrew as they were unable to maintain a committment to data collection over the length of the study period. One participant in the control group was lost to follow-up.

#### Adherence to study procedures

Prior to commencing the study, all treating nurses were assessed as competent in applying the EMLA® dressings. During the study, approximately 30 random quality assurance checks were attended with 100% compliance to intervention protocols. Consistent with current community nursing practice, eight participants performed their own dressing changes (up to three dressings each) when treating nurses or investigators were unavailable during a holiday period. For two participants, EMLA® was continued beyond the 4-week intervention period, at the patient’s request, for management of ongoing pain. EMLA® was ceased for five (16.7%) participants; one participant requested EMLA® to be ceased due to participant burden and four participants reported unchanged or increased WRP following application of EMLA®. There were confounding factors influencing WRP for the participants who reported unchanged or increased WRP such as compression therapy applied too early and severe arterial disease.

#### Data collection

Data collection instruments and time-points are presented in Table 3. The numerical rating scale (NRS) and the Wound-related Pain at Dressing Change Assessment Tool were quick and easy for both the investigator and the participant to use. The Cardiff Wound Impact Schedule was found to be long and confusing for the frailest of participants. Data from the Pain Diary was inconsistently completed and the diary was not wound specific, thus pain unrelated to CLUs was also documented by the participants. The Leg Ulcer Measurement Tool measured CLU progress; however, there was an overlap with other data collected including WRP intensity, WRP frequency and participant satisfaction with HRQoL. Health service protocols for digital photography were not always adhered to by the community nurses. This contributed to missing data, firstly due to uploading of unclear images, secondly as the computer could not calibrate some images as a ruler was not included in the image thus preventing accurate digital measurement, and finally, some images were not taken at the required time-points (47 out of 266 images). For images that were able to be measured, a specialist wound clinician was required to manually assess accuracy of CLU surface area measurements.

The mean percentage of missing data was 19% (range 8 to 25%) and was missing completely at random [11, 12]. Missing data increased as the study progressed, particularly when nurses trained in data collection were unavailable.

### Resource assessment

Human resources required to conduct this study were impacted by availability of the CI and RA who had competing full-time clinical roles. The RA was only available 1 day per week for 17 months to support the study; funding was required for the RA position.

The nurse time required for dressing change and pain data collection was the same in both groups (30 min) as was the added time taken for HRQoL and wound photography data collection at the 2, 4, 8, and 12 week time-points (1 h).

Some participants required home vists during business hours and weekends mostly due to participant frailty, lack of transport options, lack of clinic capacity, and the intervention protocol that is, daily dressings for the intervention period (weekdays: intervention group *n* = 9 (30%); control group: *n* = 6 (20%); weekends: intervention group: *n* = 30 (100%); control group *n* = 4 (33.3%): range: 1 to all visits). Seven participants required all visits in their homes (intervention group: *n* = 5 (16.6%), range: 24 to 77 visits); control group: *n* = 2 (6.6%), range: 7 to 24 visits).

A limited economic feasibility assessment was informed by a comparison of cost estimates for the intervention and usual care primary and secondary dressings. The intervention group had higher overall costs over the 12-week study period, with increased costs attributed to increased dressing frequency. Throughout the data collection period of 33 months, the intervention group required almost double the number of dressings compared to the control group (intervention group, 1232 dressings (65.4%); control group, 651 dressings (34.6%)). Daily dressings during the intervention period for the intervention group contributed to the considerable difference between groups (intervention group, 741dressings (74.6%); control group 252 dressings (25.3%). The overall cost of dressings per dressing change was less in the intervention group (intervention group, A$6.03; control group A$8.73). However, the intervention group had a 13.2% higher overall cost of primary and secondary dressing consumables over the study period (intervention group A$7441; control group A$5684), due to the increased frequency of dressings compared to the control group.

### Management assessment

Participants were initially seen in the community health service clinics. However, as the study progressed, 13 participants had difficulty attending the clinics for treatment. Hire cars were provided for these participants as a short-term solution however, due to budget restrictions, this strategy was not feasible and was discontinued after 20 months. Subsequently, participants unable to attend the clinic were treated in their homes during business hours and weekends as previously reported using existing community nursing resources. This meant that some visits were attended by nurses not educated in the intervention protocols at the beginning of the study; nurse continuity was also an issue. The potential impact on the data collected during home visits was anticipated; the CI attended the visit particularly on weekends or made phone contact with the nurse to explain the protocols and procedures prior to the home visit.

Participant burden was observed in this study. The length of the eligibility interview, randomisation and recruitment processes (1 to 3 h), the length of the intervention (4 weeks) and study periods (12 weeks), the length of some data collection tools (up to 50 questions), the frequency of the data collection (at each dressing change), the average age and health status of the participants (73 years) and requirement of participants to come to the clinics, all contributed to participant burden.

### Scientific assessment

There was no difference in WRP intensity scores between groups before dressing change over the 4-week intervention period (intervention group: mean 4.10 [95% CI 3.55, 4.63] vs control group: mean 4.21 [95% CI 3.66, 4.76]). Nevertheless, there was a statistically significant reduction in WRP for the intervention group compared to the control group during dressing change (intervention group: mean 3.39 [95% CI 2.59, 4.19] vs control group: mean 4.82 [95% CI 3.98, 5.66]) and after dressing change: (intervention group: mean 2.71 [95% CI 1.99, 3.43] vs control group: mean 3.92 [95% CI 3.16, 4.68]) [11]. EMLA® was tolerated well for 4 weeks by 83.3% (*n* = 25) of the intervention group. The remaining 16.6% (*n* = 5) of the intervention group had adverse effects from the application of EMLA® to their leg ulcers. Erythema, pallor, itching, oedema, purpuric or petechial lesions, or allergic reaction were not reported by the attending clinicians; however, five participants required EMLA® to be ceased due to increased or unchanged WRP and increased wound size. Usual care was recommenced on all participants. There were no serious adverse events to the intervention during this study.

Interestingly, two participants required recommencement of EMLA® following the 4-week intervention period at their request for the remainder of the 12 week study period due to significant exacerbation of their WRP after cessation of the EMLA® and commencement of usual care. Once EMLA® was recommenced, WRP was reduced.

## Discussion

This is the first study to investigate EMLA® used as a primary dressing for relieving wound-related pain for patients with painful chronic leg ulcers. The pilot study was pivotal to assessing feasibility for a larger clinical trial and to determine potential effectiveness. The identification of potential practical problems that may cause breakdown when implementing the research study protocol into clinical practice is crucial for the success of a larger study [31, 32]. By undertaking this feasibility study, we have been able to identify ways in which the study protocol for a future multicentre randomised controlled trial could be refined although the generalisability of our results may be limited due to participant enrolment from a single site. Solutions to manage any challengers during the study and recommendations for protocol modifications to inform a larger RCT are presented in Table 5. The key learnings from this pilot study related to recruitment and retention of participants, establishing resources required and managing data collection to ensure data accuracy and completeness and are presented below.

**Table 5:** Feasibility challenges, solutions and recommendations

	Challenges	Solutions and recommendations
Feasibility		
Recruitment rate	- Recruitment was protracted. The reasons were: - Insufficient dedicated research personnel coupled with competing full-time workloads - Structured screening process prevented identification of all eligible patients - Exclusion criteria too restrictive	Solutions: - Employment of an RA - Community nursing referral screening tool was developed - Transportation was provided for some participants to clinics - Amendments to some exclusion criteria - Some participants were treated in their homes
Retention	- Participant burden was increased for some frail participants - There was limited availability of transportation to clinics	Recommendations: - Employment of a dedicated trial manager - A comprehensive screening process to identify potential participants at the beginning of the study - Establish centralised intake system to identify potential patients at first CN contact - In-depth review of nursing resources including skill mix prior to commencement of study - Include home visits for treatments in a larger study
Resources	- Insufficient human resources to conduct the research within the timeframe - Poor continuity of nursing services especially for home visits - Some patients could not attend clinics - Re-calibration of photo digital planimetry software required for wound measurement accuracy	Solutions: - Further institution support was acquired during study - Support from experienced clinical nurses to administer intervention and collect data - The application of the intervention was able to be accommodated within existing clinic schedules - Information technology support was acquired Recommendations: - Review treatment location options - Collaboration with health service management regarding staff backfill to promote continuity - Study-specific investigators and support staff - Usual care wound products for multisite larger study as per health service formulary; negotiate for in-kind supply of wound products for a multisite RCT - Use dedicated study equipment
Management	- Oversight of the study was difficult for CI and RA due to competing full-time workload - Participant burden was high - Prolonged consent, randomisation and baseline data collection processes - Poor quality photos of some wounds - Existing resources made available by health service for wound size measurements were insufficient - Research data were collected parallel to health service data resulting in some duplication and extended nurse time - Data collected by clinicians untrained in study processes resulted in higher rates of missing data	Solutions: - Ensured computer software for data entry and analysis available and appropriate - Ensured technology to acquire, store and measure wound photography appropriate - Separated recruitment processes from baseline data collection - Established secure data storage - There was easy access to information technology support - Wound size measurements by wound specialist nurse Recommendations: - Trial-specific investigators, data collectors and administrative team with no competing interests - Shorten recruitment processes - Revise and reduce the size and number of data collection tools and data collection time points
Scientific	- Change to intervention protocol required for some participants due to negative clinical response	Solutions: - Intervention ceased, changed to usual care Recommendations: - Investigate potential use of placebo

*RA* research assistant, *CI* chief investigator, *CN* community nursing

In terms of the feasibility objectives, although we were able to recruit 100% of the target sample, it was not achieved in the predicted timeframe of 12 months. We were able to meet participant retention and intervention adherence targets of 80%, and the study outcomes suggest that it is feasible to proceed to a larger multisite clinical trial to examine EMLA® as a primary dressing on painful CLUs. However, modifications to the protocol are recommended.

### Recruitment

Participant and research process factors influenced recruitment rates in this study. This experience is not uncommon in RCTs [33] where up to two-thirds of trials are unable to successfully recruit their original target [34, 35]. Protracted or ineffective recruitment can have undesirable scientific, ethical and economic consequences [36, 37]. Although random assignment may result in refusal to participate in a study [36, 38], this was not the case in this study. We overestimated the pool of patients with CLUs in the community nursing service that would meet the eligibility criteria. Known as ‘Lasagna’s Law’, this phenomenon is a common problem in clinical research with the evidence indicating that the incidence of the disease investigated reduces to 10% of the original estimate once the study starts [38]. This is a common threat to the success of clinical research resulting in increased direct costs and challenges the commitment and morale of research staff and participants [38]. Study processes and under resourcing of research personnel contributed to slower than expected recruitment, and it is likely that eligible patients were missed.

Lack of interest, inability to commit, physical and time limitations, change to daily activities and inability to travel to the community nursing clinics were patient-related factors affecting slow recruitment in this study; all of which are frequently cited in the literature [38]. The biggest obstacle to recruitment was patient eligibility at initial screening; we excluded more than we had predicted (34%), thus potential participants with painful CLUs were omitted from the study which may have negatively affected the generalisability of our results. A literature review found that an average of 30% of patients attending eligibility screening in RCTs are ineligible [34]. The majority of potentially eligible participants in this study were older; this was expected since the older person is more likely to succumb to CLUs [39–41].

Considering the prevalence of chronic leg ulcers in society, most chronic wound trials have small sample sizes reflecting the difficulty in recruiting patients if the eligibility criteria are too restrictive [42]. It became apparent that two of the exclusion criteria were considered unnecessary hence amendments were made to the exclusion criteria to increase the recruitment rate previously described. Based on these findings, we acknowledge the importance of understanding the needs and abilities of the prospective study population prior to developing eligibility criteria for a larger study. Prior to recruitment, we over-estimated how many of the potentially eligible patients would qualify for the study. Although recruitment was initially slow, the rate improved when adequate resources for screening and recruitment were in place.

The difficulty in recruiting older individuals has been well identified; however, age itself does not determine an individual’s ability to give consent to research [43]. There are factors however that are associated with age that could impact on an individual’s ability to consent effectively such as frailty, fatigue, cognitive impairment, chronic disease and/or feelings of vulnerability [43]. Nevertheless, in this study, we had a good consent rate (86%) when compared to other RCTs relating to CLUs [44–46].

### Community nurses

Most wound care is attended to in the community setting and community nurses are essential for the identification of potential participants in wound care research. They have been described as “effectively the gatekeepers to trial participation” [47]. They are an important link between the investigators and participants and can influence recruitment and retention rates [38]. In this study, community nurses were enthusiastic about being involved in an RCT and could see the benefits to themselves and to the patient. They were provided with criteria to assist them to identify potentially eligible patients however, subsequently, the demands of their clinical workload impacted their ability to undertake patient screening and contributed to delayed or missed participant identification [47].

### Chronic leg ulcer types

Patients with venous, arterial, mixed (arterial/venous) and diabetic foot ulcerations were recruited so outcomes reflected real-world clinical practice. These ulcer types differ in their underlying aetiology and wound characteristics. To increase the ability to meet our required sample size, patients with any of the above CLU types were included. We acknowledge that such heterogeneity can threaten study validity and usefulness of a clinical trial [42]. Subgroup analysis would be a solution however, a much larger sample size would be required [42] which was not realistic for this single-centre pilot study. Additionally, simple randomisation may not be sufficient to provide well-balanced treatment groups regarding confounders in this broadly defined study; a large, multicentre RCT using stratified randomisation may be appropriate [42].

### Attrition

To maintain statistical power, we aimed for less than 20% loss to follow-up. Attrition rates of 20% or more introduces bias and is a serious threat to the internal and external validity by altering the structure of the intervention and control groups [38, 48]. Common predictors for study withdrawal are older age and functional impairment [49]. The attrition rate for this study was only 10% which was encouraging considering the majority of participants were older, frail, in significant pain, had committed to a long study period, were subject to a large amount of data collection and were required to travel to community nursing clinics. To aid retention, some participants required home visits; however, in a larger study, this will require more time allocation plus additional costs [49].

### Participant burden

Participant burden is a subjective, multidimensional construct relating to the perception of the participants physiological, physical and/or economic adversity with involvement in the research process [50, 51]. Investigators have traditionally addressed participant burden in clinical trials by focusing on direct risks associated with the intervention or data collection procedures. Nevertheless, it is the indirect burden that can vary due to factors such as study duration, intensity and invasiveness of study procedures [50] that needs to be considered. This pragmatic pilot study has identified direct and indirect factors that contributed to participant burden and will be able to inform a larger study to use a more pragmatic approach to reduce participant burden to maximise research participation and response rates (Table 5).

### Missing data

In this study, we had a mean percentage of 19% missing data. The literature does not identify an established cut-off regarding an acceptable percentage of missing data; 5% or less is considered inconsequential, and more than 10% can result in a biased statistical analysis [52]. Additionally, missing data mechanisms and patterns can have a bigger influence on results than the proportion of missing data [52]. To manage missing data in this study, we attempted to follow up all participants, included all available data in the analysis making a plausible assumption about missing data and did a sensitivity analyses that weakened the assumptions about missing data [48]. Missing data was missing completely at random; therefore, systemic attrition did not occur and an unbiased treatment effect estimate was derived from the obtained data [48, 53]. For a definitive effectiveness trial, missing data would need to be minimised to reduce the threat to study validity. Evidence shows that 95% of RCTs report some missing data which can threaten the validity of an RCT, make a true intention to treat analysis difficult to achieve, reduce the power and efficiency of the study and lead to bias [48].

### Strengths of the study

The strengths of the study include the recruitment of 100% of the target population, retention of 90% of our sample, assessment of the fidelity of the intervention, inclusion of objective outcome measures, and the ability to refine protocols and procedures. Generalizability of the results of this pilot study may be limited due to participant enrolment from a single health service. Consequently, context-specific issues that may be influenced by local, regional or country specific practices are unknown. Furthermore, there were fewer eligible participants than initially anticipated. The exclusion criteria may have resulted in some patients with painful CLUs being overlooked for inclusion in this study.

Bias could have been introduced since the participants, treating nurses and researchers could not be blinded to the intervention. Additionally, process evaluation was not attended by a neutral party but by the researchers themselves; therefore, there is the potential that further biases may have been introduced. The study protocols and procedures placed considerable demands on the mostly frail aged participants, the treating nurses and health service resources all contributing to missing data. We recognise the need to minimise the difficulties identified in this study that participants and investigators may encounter when designing a protocol for a larger multisite study. Furthermore, regulations for the use of EMLA® on open wounds such as CLUs and its drug schedule status would have to be ascertained prior to an international study as these factors may differ between countries.

## Conclusion

Our goal is to move towards a larger study with wound-related pain as the primary endpoint conducted on individuals with painful chronic leg ulcers. This pilot study provides important feasibility information that can be used to inform a definitive future study. In the interim, this study provides insight into the potential effectiveness of EMLA® on painful chronic leg ulcers, wound healing and health-related quality of life when used as a primary dressing.

## Abbreviations

CI: Chief investigator
CLU: Chronic leg ulcer
DSMB: Data safety monitoring board
EMLA®: Eutectic Mixture of Local Anaesthetics
GCP: Good Clinical Practice
HREC: Human Research Ethics Committee
HRQoL: Health-related quality of life
RA: Research assistant
RCT: Randomised controlled trial
WRP: Wound-related pain
